# Sub-inhibitory concentrations of some antibiotics can drive diversification of *Pseudomonas aeruginosa* populations in artificial sputum medium

**DOI:** 10.1186/1471-2180-13-170

**Published:** 2013-07-23

**Authors:** Elli A Wright, Joanne L Fothergill, Steve Paterson, Michael A Brockhurst, Craig Winstanley

**Affiliations:** 1 Institute of Infection and Global Health, University of Liverpool, The Ronald Ross Building, 8 West Derby Street, Liverpool, L69 7BE, UK; 2 NIHR Biomedical Research Centre in Microbial Disease, University of Liverpool, Liverpool, UK; 3 Institute of Integrative Biology, University of Liverpool, Liverpool, L69 7BE, UK; 4 Department of Biology, University of York, York, YO10 5DD, UK

**Keywords:** Antibiotics, Cystic fibrosis, Population biology, *Pseudomonas aeruginosa*

## Abstract

**Background:**

*Pseudomonas aeruginosa* populations within the cystic fibrosis lung exhibit extensive phenotypic and genetic diversification. The resultant population diversity is thought to be crucial to the persistence of infection and may underpin the progression of disease. However, because cystic fibrosis lungs represent ecologically complex and hostile environments, the selective forces driving this diversification *in vivo* remain unclear. We took an experimental evolution approach to test the hypothesis that sub-inhibitory antibiotics can drive diversification of *P. aeruginosa* populations. Replicate populations of *P. aeruginosa* LESB58 were cultured for seven days in artificial sputum medium with and without sub-inhibitory concentrations of various clinically relevant antibiotics. We then characterised diversification with respect to 13 phenotypic and genotypic characteristics.

**Results:**

We observed that higher population diversity evolved in the presence of azithromycin, ceftazidime or colistin relative to antibiotic-free controls. Divergence occurred due to alterations in antimicrobial susceptibility profiles following exposure to azithromycin, ceftazidime and colistin. Alterations in colony morphology and pyocyanin production were observed following exposure to ceftazidime and colistin only. Diversification was not observed in the presence of meropenem.

**Conclusions:**

Our study indicates that certain antibiotics can promote population diversification when present in sub-inhibitory concentrations. Hence, the choice of antibiotic may have previously unforeseen implications for the development of *P. aeruginosa* infections in the lungs of cystic fibrosis patients.

## Background

The mutations that lead to the genetic disorder cystic fibrosis (CF) predispose patients to chronic bacterial lung infections, particularly with the opportunist *Pseudomonas aeruginosa*[1]. Once established, these chronic bacterial infections are virtually impossible to eradicate and lead to a decline in pulmonary function, reduction in quality of life and premature death [2-4]. During chronic lung infections in CF patients, *P. aeruginosa* populations accumulate mutations generating considerable population diversity, leading to both genotypic and phenotypic variations [5-9]. This diversification process can lead to various phenotypic sub-types co-existing in the same population, varying in characteristics such as colony morphology, including mucoid conversion, the inactivation of quorum-sensing (QS) and other virulence-associated traits, hypermutation, loss of the O-antigen components of the lipopolysaccharide, loss of motility, resistance to antibiotics and changes in nutritional requirements [7,10-15]. In a previous study, we analysed 1720 isolates of the Liverpool Epidemic Strain (LES) of *P. aeruginosa* from 43 sputum samples obtained from 10 chronically infected adult CF patients [9]. Following the characterisation of the isolates for 15 traits, 398 haplotypes (defined as a specific combination of genetic and phenotypic traits) of the LES were identified. The majority of phenotypic diversity occurred within individual CF patients. We further showed that this diversity was highly dynamic, with a rapid turnover of subtypes over time.

Certain phenotypic changes, such as the evolution of hypermutability and mucoidy, are commonly reported in CF isolates of *P. aeruginosa* and, therefore, suggest conserved evolutionary pathways of adaptation [16,17]. The CF lung presents a highly complex environment that is viscous, spatially heterogeneous and compartmentalized. Moreover, it houses a rich microbiota of coexisting species, which may compete for resources or cause *P. aeruginosa* mortality (e.g., bacterial killing via bacteriocins or bacteriophages). Furthermore, the CF lung environment exposes colonising bacteria to physiologically stressful conditions, including host immune responses, oxidative stress and antibiotic treatment [18,19]. Thus it has been hypothesised that phenotypic diversification allows *P. aeruginosa* to adapt to the hostile environment of the CF lung thereby enabling long-term persistence. Moreover, it has been argued that such diversification leads to either increased or reduced virulence [16,20] and could therefore be crucial to understanding disease progression and treatment. While all of these facets of the CF lung environment could potentially play a role in mediating the diversification of *P. aeruginosa*, it is not possible to disentangle or determine the relative importance of these selective forces *in vivo*. A powerful approach to understanding the contribution of particular selective forces to driving bacterial diversification is through experimental evolution, whereby replicate populations are exposed to defined selective conditions in the laboratory.

CF patients are typically subject to extended antibiotic regimes, but the drugs do not necessarily reach the entire lung at inhibitory concentrations [21]. Therefore, sub-inhibitory antibiotic exposure could be one factor that promotes *P. aeruginosa* diversification in the CF lung. Consequently, a better understanding of the responses of *P. aeruginosa* populations to these sub-inhibitory concentrations of antibiotics in the CF lung would allow clinicians to make better informed choices of antibiotic regimes.

Although it is likely that most CF patients acquire *P. aeruginosa* infections from diverse environmental reservoirs and thus carry their own unrelated strains, several multidrug-resistant “epidemic” strains capable of patient to patient transmission have been identified [22]. The LES is the most widespread transmissible strain of *P. aeruginosa* in the UK [23], and has also been reported in North America [24]. It has been detected in as many as 79% of adult CF patients in a Liverpool CF centre [25]. The high prevalence of LES in CF patients is a concern, given that chronic LES infection has been associated with a greater deterioration in pulmonary function and nutritional state [26] and increased antibiotic resistance [27]. In this study, we analysed *P. aeruginosa* LES populations in an artificial sputum medium (ASM) model that mimics CF sputum in terms of composition. Various groups have utilised ASM models to study, for example, gene expression patterns and the effects of bacteriophages [28-30]. *P. aeruginosa*, when cultured in ASM, forms biofilms and diversifies with respect to phenotype, in a manner that resembles behaviour in the CF lung [30]. We hypothesise that exposure to sub-inhibitory concentrations of antibiotics will drive bacterial diversification, possibly through a combination of antibiotic-induced mutagenesis or through the regulation of gene transcription [31-36]. Consequently, the objective of this study was to test the hypothesis that exposure to sub-inhibitory concentrations of antibiotics has a role to play in promoting *P. aeruginosa* population diversification during growth in an ASM model.

## Results

### Sub-inhibitory antibiotics promote diversification of *P. aeruginosa* LESB58

**Figure 1:**
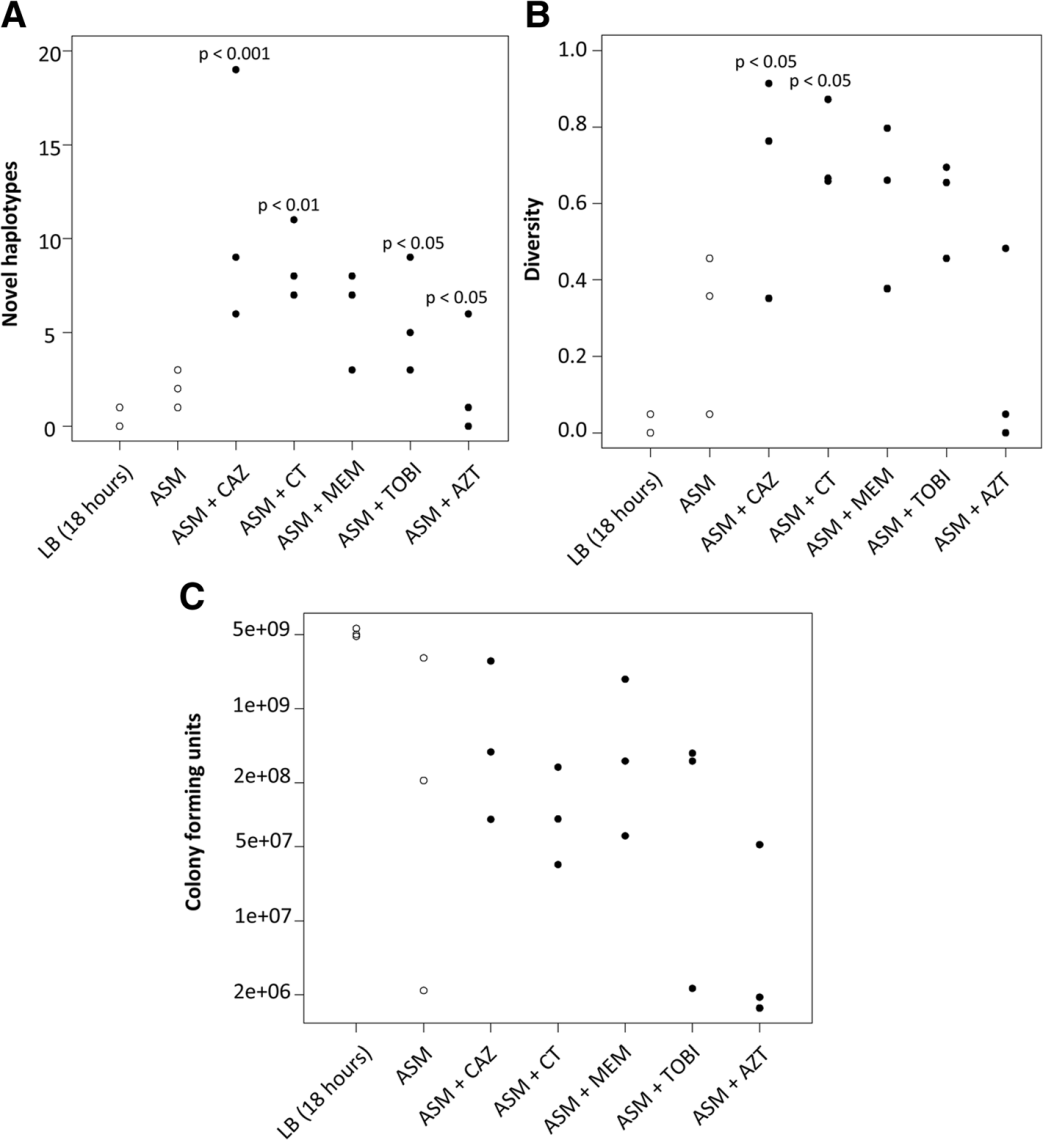
**Diversification of LESB58 grown in the presence (closed circles) or absence (open circles) of antibiotics.** Forty isolates of LESB58 from each culture were characterised using 13 traits (colony morphology, pyocyanin production, hypermutability, auxotrophy, susceptibility to 6 antibiotics and the presence/absence of 3 genomic regions). Therefore, 120 isolates were analysed for each experimental and control group across the 3 replicate populations. Isolates with different traits were identified as being a different haplotype. 3 replicate populations from each of the following treatments were analysed: LB (18 hours)**,** ASM**,** and ASM with ceftazidime (+ CAZ), ASM with colistin (+CT), ASM with meropenem (+MEM), ASM with tobramycin (+TOBI), ASM with azithromycin (+AZT). **(A)** Number of novel haplotypes found within each replicate population. **(B)** Haplotype diversity found within each replicate population, defined as the probability of two randomly picked haplotypes being non-identical. **(C)** The colony forming units found within each replicate population following culture. P-values represent comparisons with ASM alone.

**Figure 2:**
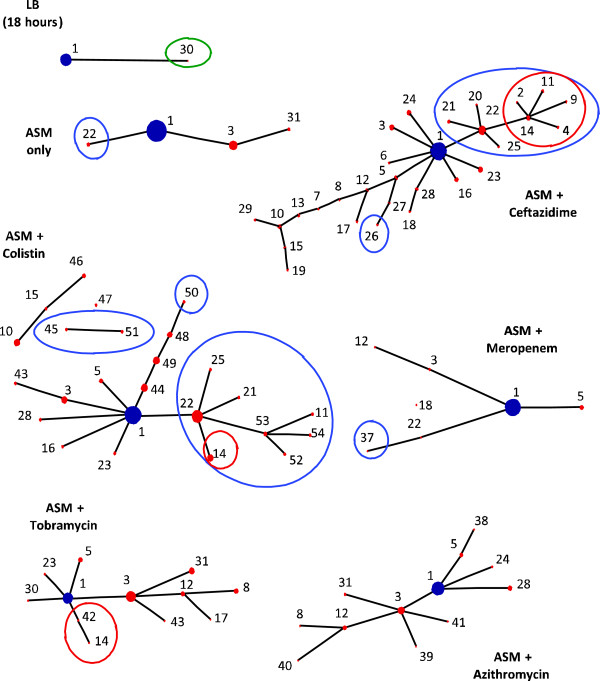
**Population structure of LESB58 grown in ASM with and without sub-inhibitory concentrations of antibiotics.** Each population structure of LESB58 was calculated using 13 traits (colony morphology, pyocyanin production, hypermutability, auxotrophy, susceptibility to 6 antibiotics and the presence/absence of 3 genomic regions) for the total 120 isolates by the eBurst algorithm. Each dot (and subsequent number) represents one novel haplotype, with dot size reflecting abundance. The larger the dot size, the more abundant that novel haplotype was in the 120 isolates that we characterised. Haplotypes designated with the number 1 represent isolates with the same characteristics as the *P. aeruginosa* LESB58 wild-type. The haplotypes representing isolates that had a straw-coloured colony morphology are circled in red; the haplotypes representing isolates that did not over-produce pyocyanin are circled in blue; and the one isolate that was hypermutable is circled in green. The remaining haplotypes differed in their antibiotic susceptibility profiles (All groups: n = 120).

The emergence of novel haplotypes was observed in all culture conditions, but the presence of sub-inhibitory concentrations of certain antibiotics significantly increased both the number of novel haplotypes (p <0.01, LRT = 48.8, d.f. = 6) and the haplotype diversity found within populations (p < 0.01, F_6,14_ = 5.90) relative to control populations (Figures 1 and 2). However, some antibiotics contributed to this diversity more than others. Diversification was highest in the presence of ceftazidime and colisitin (total of 29 and 25 novel haplotypes, respectively) compared to azithromycin (total of 12 novel haplotypes), tobramycin (total of 12 novel haplotypes) and meropenem (total of 7 novel haplotypes; Figures 1 and 2). Furthermore, based on mean antibiotic resistance across the antibiotics tested, the Brown-Forsythe-Levene test of equality of variances between 7 groups gave a test statistic of F_(6,833)_ = 15.3, p < 0.001. Exposure to ceftazidime and colistin gave a high variance, and the differences between means are statistically significant (F = 61.5, P < 0.001). There was no significant difference in the colony forming unit (CFU) values between the populations exposed to antibiotics in ASM and in populations exposed to ASM alone. ASM appears to generate variation in bacterial numbers among replicates.

### Haplotype properties differ between different antibiotic exposures

**Figure 3:**
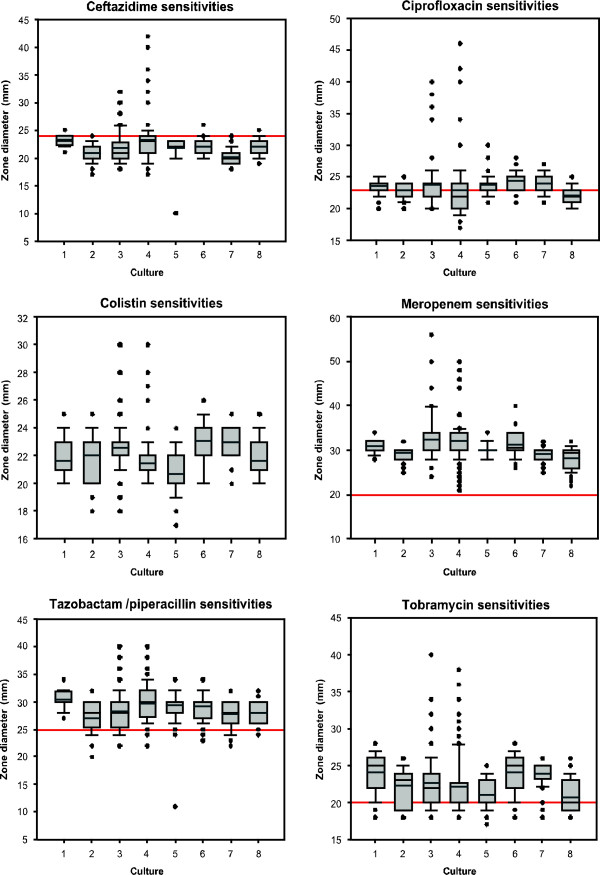
**Variations in zones of inhibition within LESB58 populations.** The 120 LESB58 isolates obtained from the triplicate ASM cultures were assessed for susceptibility to six commonly used antibiotics (ceftazidime, ciprofloxacin, colistin, meropenem, tazobactam/piperacillin and tobramycin). Boxplots showing the range in the diameter of the zones of inhibition to these antibiotics are presented. 1. LB (18 hours) 2. ASM 3. ASM with ceftazidime 4. ASM with colistin 5. ASM with meropenem 6. ASM with tobramycin 7. ASM with azithromycin 8. Normal range of LESB58 (Groups 1–8: n = 120). The red line represents the cut-off for the sensitivity of *P. aeruginosa* to the antibiotics tested, in accordance with the guidelines of Andrews and Howe [37]. The values above the red line denote a higher sensitivity to antibiotics and the values below the line denote a higher resistance.

Diversification of *P. aeruginosa* LESB58 in ASM cultured with and without the various antibiotics was observed only with respect to colony morphology, pyocyanin production and antibiotic susceptibilities (Table 1). The culture of LESB58 in ASM with sub-inhibitory concentrations of ceftazidime and colistin led to diversity in antimicrobial susceptibilities, changes in colony morphology and a loss of pyocyanin production (Table 1). LESB58 cultured in the presence of these antibiotics, generated more isolates that were outside the normal range of the antibiotic sensitivity profiles of LESB58 controls (Figure 3). In addition, exposure to azithromycin and tobramycin promoted increased cross-resistance to other antibiotics (Table 1, Figure 3). There was no variation in the auxotrophic phenotype in the isolates analysed in all experimental and control groups (LESB58 has an auxotrophic phenotype). The populations exposed to meropenem exhibited no clear phenotypic diversification (Table 1 and Figure 2).

**Table 1:** Number of isolates in each group (total of 120) exhibiting each of the traits measured

	**Colony morphology**		**Virulence**	**Mutations**	**Outside normal range of antimicrobials susceptibility**					
**Culture**	**Green non-mucoid**	**Straw non-mucoid**	**Pyocyanin**	**Hypermutability**	**Ceftazidime**	**Ciprofloxacin**	**Tobramycin**	**Meropenem**	**Colistin**	**Tazobactam/piperacillin**
ASM	120	0	117	0	3	0	19	0	2	8
ASM + CAZ	110	10	92	0	16	19	20	18	10	11
ASM + CT	113	7	84	0	17	37	29	15	7	9
ASM + AZT	120	0	120	0	0	16	34	0	4	4
ASM + MEM	120	0	118	0	1	8	4	0	0	1
ASM + TOBI	118	2	119	0	1	24	69	3	22	1
LB (18 hours)	120	0	120	1	0	0	0	0	0	0

Isolates that were characterized as being outside the normal range of antimicrobial susceptibility typically observed in LESB58, included isolates that had either an increased or reduced susceptibility to the antibiotic under test. ASM = Artificial Sputum Medium, LB = Luria Bertani, CAZ = Ceftazidime, CT = Colistin, AZT = Azithromycin, MEM = Meropenem and TOBI = Tobramycin.

Furthermore, there was no variation in the carriage of the LES prophage 5, LES prophage 2 and LES Genomic Island (GI)-5 in the isolates analysed (all isolates tested contained these genetic elements). None of the tested isolates grown in ASM (from both treatment and control groups) displayed the hypermutable phenotype. The only hypermutable isolate detected in this study was generated following growth in Luria Bertani (LB) for 18 hours (Figure 2 and Table 1). Although diversification occurred with respect to only a few of the phenotypic properties tested, the proportions of the isolates exhibiting these traits varied considerably between treatment groups (Figure 1). The proportions of these phenotypic changes accounted for the within and between-treatment group variation seen in the numbers of mutant haplotypes (Figure 1). Hierarchical analysis of variance indicated that the majority (77%) of diversity was distributed between isolates within populations, rather than the same traits systematically apportioned between replicate populations or between treatments (Table 2).

**Table 2:** **Hierarchical analysis of variance (σ**^**2**^**) for diversity**

	**Sigma**	**%**
Variations between treatment	0.03	6.18
Variations between samples within treatment	0.09	16.42
Variations within samples	0.42	77.40
Total variations	0.54	100.00

## Discussion

Although it is known that the phenotypic and genotypic characteristics of *P. aeruginosa* populations within the CF lung fluctuate over time [9,16], the factors that are responsible for this diversification are not fully understood. When *P. aeruginosa* LESB58 was grown in ASM with and without sub-inhibitory concentrations of antibiotics, we observed differential effects of antibiotics commonly used to treat CF patients on the diversity of LESB58 populations in the ASM model. In particular, increased levels of phenotypic diversification occurred in LESB58 populations grown in ASM when sub-inhibitory concentrations of colistin, ceftazidime and azithromycin were present. However, extensive diversification of the *P. aeruginosa* populations was not seen in the presence of sub-inhibitory concentrations of meropenem.

There are a number of mechanisms by which sub-inhibitory concentrations of antibiotics could potentially enhance bacterial diversification. One potential mechanism could involve the antibiotics inducing mutagenesis within bacterial populations, causing variation and/or promoting the hypermutability phenotype [31-34]. A second potential mechanism could involve the antibiotics acting as signalling molecules, altering the QS systems within bacterial populations and subsequently promoting social evolution and diversification [35,36,38]. Antibiotic exposure has been shown to induce mutagenesis by triggering the SOS response and thus increasing the expression of error-prone DNA polymerases, which could give rise to diversity within bacterial populations [31-34]. It is possible that ceftazidime induced mutagenesis in the LESB58 populations through the induction of the SOS response. It has been suggested that this increase in mutagenesis is an adaptive strategy that favours the acquisition of antimicrobial resistance and survival in harsh environments [33,39]. However, it has been argued that the generation of genetic variants within the CF lung does not require the SOS response, and that starvation and oxidative stress caused by antibiotic exposure can promote diversity within *P. aeruginosa* biofilms [31,40-42].

The hypermutable phenotype occurs as a consequence of defects in error avoidance or DNA repair genes, typically termed anti-mutator genes [43]. It has been suggested that hypermutability, promoted by extrinsic and intrinsic factors, is the driver of *P. aeruginosa* adaptation and survival in the CF lung [44,45]. Although phenotypic diversification of LESB58 was observed following culture in ASM, especially when sub-inhibitory concentrations of colisitin, ceftazidime or azithromycin were present, no hypermutable isolates were detected. In our previous study using LES isolates from multiple CF patients, we found hypermutable sub-types but only at low frequency [9]. In this study we found no evidence that hypermutability was driving this diversification and adaptation process. This supports work by Ciofu *et al.*[10] who found that the hypermutability phenotype was not essential for the acquisition of mucoidy and loss of QS. Other studies have also suggested that spontaneous mutation and mutator strains are not required to produce genetic variants in populations of *P. aeruginosa* within the CF lung [40,46].

It has been shown that sub-inhibitory concentrations of antibiotics can act as signalling molecules that regulate bacterial gene transcription, physiology and virulence [36,38,47-51]. In particular, tobramycin, colistin and azithromycin at sub-inhibitory concentrations have been shown to modulate the QS networks in bacterial populations [35,36,38]. These antibiotics are commonly used to treat CF patients and, therefore, the signalling activities of these antibiotics could increase bacterial fitness for survival in the harsh environment of the CF lung [38], suggesting that the classical view of antibiotics acting only to reduce bacterial fitness and virulence is not always the case. In the current study, across all the ASM cultures, no single dominant phenotypic variant emerged. Some patterns in the diversification process were evident. For example, isolates lacking the pyocyanin production phenotype occurred following culture in ASM with ceftazidime or colistin. However this was only evident in two out of the three biological replicates (ASM + Ceftazidime: 27.5% and 40% of the isolates; ASM + Colistin: 42.5% and 40% of the isolates), highlighting the variability between replicates. A previous study by Cummins *et al.*[38] has shown that sub-inhibitory concentrations of colisitin actually increases pyocyanin production. Pyocyanin production is regulated by QS, which relies upon small diffusible signal molecules interacting with transcriptional activators to couple gene expression with cell population density. Although QS-deficiency is a common feature amongst *P. aeruginosa* CF isolates [16,52,53], QS regulates a number of factors of relevance to CF, including pyocyanin and LasA production [54]. Our previous studies suggested that LES populations in CF comprise a mixture of QS-positive and QS-deficient bacteria [7,9,54], which is what we have observed in this study in ASM. The QS-deficient populations could benefit at the cost of QS-positive populations.

The main phenotypic variations involved changes in colony morphology, pyocyanin production and antimicrobial susceptibilities. A high diversity in the antimicrobial susceptibility profiles of CF isolates within adult sputum samples has been demonstrated previously [9], highlighting the limitations of performing antimicrobial susceptibility tests on a single isolate from a CF patient sputum sample. It was also shown that using one antibiotic could lead to enhanced resistance to a different, unrelated antibiotic [9]. A similar pattern was observed in this study, when exposure to one antibiotic altered the antibiotic susceptibility profiles to unrelated antibiotics. In particular, exposure to azithromycin, tobramycin or ceftazidime led to an increase in resistance to tazobactam/piperacillin. This could have serious clinical consequences for the CF patient, in terms of the generation of antimicrobial resistant *P. aeruginosa* populations, because CF patients are regularly exposed to a number of different antibiotics. In our study, the presence of meropenem had a weaker effect on diversification compared to the other antibiotics, despite having a similar mechanism of action to ceftazidime. However, it is possible that cell death was occurring in these populations, since the numbers of cells obtained following culture were generally lower. This is despite the meropenem concentration in ASM being 8-fold less than the minimum inhibitory concentration of this antibiotic. Therefore, the apparent reduction in diversity could be attributed to the populations exhibiting cell death. This suggests that there may be a clinical advantage to using some antibiotics (eg. meropenem) rather than others. It would also be interesting to analyse combinations of two antibiotics, since it is often the case that dual therapy is used clinically. The identification of individual mutations within the LESB58 populations to explain the changes in individual phenotypic traits would have been beyond the scope of this work.

## Conclusions

This study suggests that the exposure to sub-inhibitory concentrations of certain antibiotics can drive phenotypic diversification of *P. aeruginosa* populations in the ASM model. This may help to explain the observed diversification of *P. aeruginosa* in natural CF lung infections, although other factors such as the host immune response, other members of the microflora, or bacteriophages may also contribute. Understanding *P. aeruginosa* diversification in the CF lung could alter the way we control these infections, in particular during the early stages. Diversification of the *P. aeruginosa* populations in the CF lung, and the emergence of phenotypes such as mucoidy, are signs of adaptation leading to a chronic infection state. Diversification may also lead to enhanced antimicrobial resistance. Antibiotics that do not cause extensive diversification might be utilised to prevent diversification, and possibly slow down the development of a chronic infection state. Therefore, being able to delay, control or possibly reduce diversification could be advantageous for the CF patient. This could also be achieved by using antibiotics that permeate the lung and the bacterial biofilms better to achieve inhibitory concentrations, but it could also be important to choose antibiotics that do not promote diversification. Hence a better understanding of the differential effects of various antibiotics on diversification of *P. aeruginosa* populations could provide valuable information to help clinicians choose the best antibiotics for CF patients.

## Methods

### ASM preparation and culture conditions

**Figure 4:**
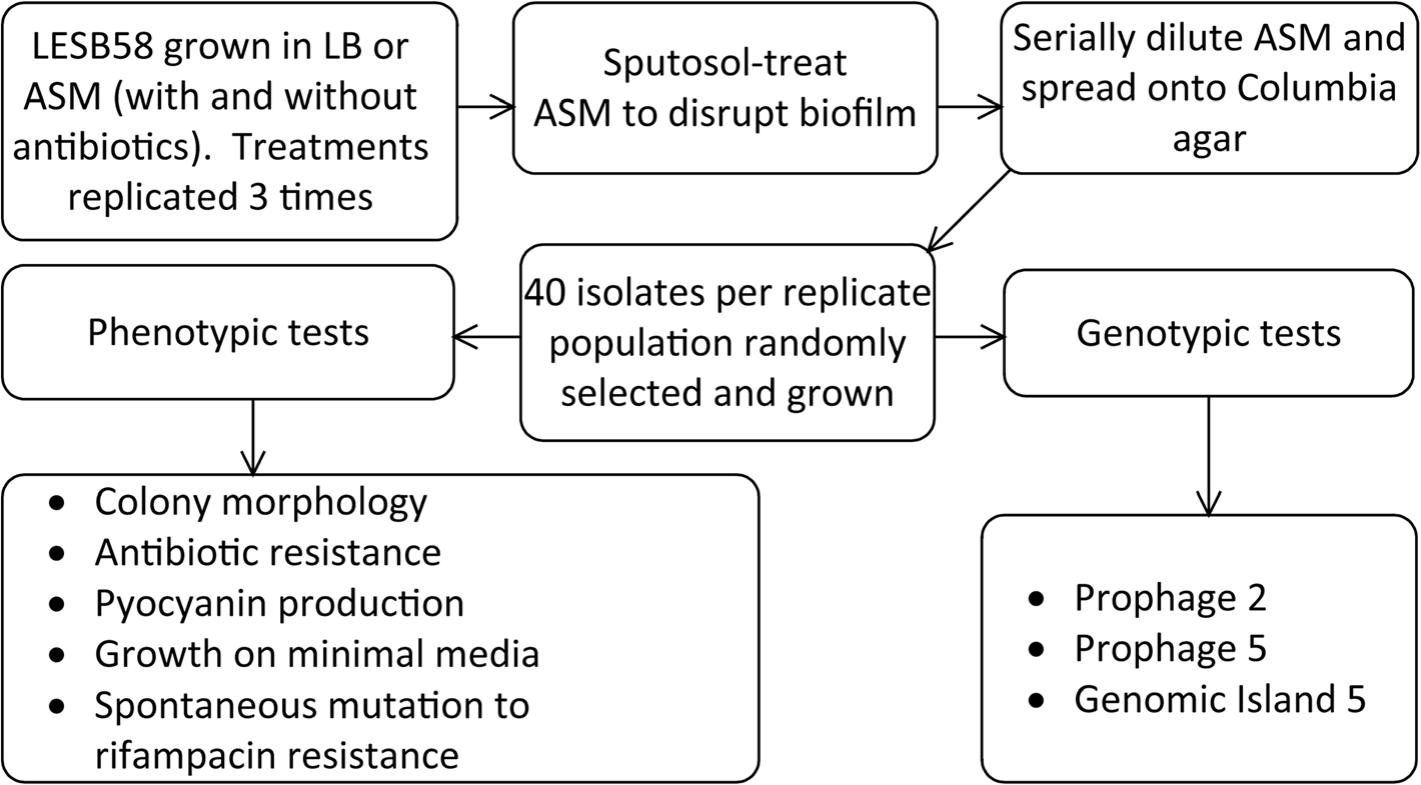
**Summary of experimental design.** The figure describes the steps involved in processing of the LESB58 populations cultured in ASM, with or without antibiotics, and the phenotypic and genotypic tests performed on individual isolates.

The ASM was prepared following the protocol of Sriramulu *et al.*[30] and Kirchner *et al.*[55]. ASM contains mucin from porcine stomach (Sigma-Aldrich, Gillingham, UK), DNA (Sigma-Aldrich), the iron-chelator diethylene triamine pentaacetic acid (Sigma-Aldrich), NaCl (Sigma-Aldrich), KCl (Sigma-Aldrich), egg yolk emulsion (Sigma-Aldrich) and all essential and non-essential amino acids (Fisher Scientific, Loughborough, UK and Sigma-Aldrich) at concentrations found in an average CF patient [30]. A single colony of the genome-sequenced *P. aeruginosa* CF isolate LESB58 [56] was used to inoculate LB broth and cultured for 18 h at 37°C and 200 rpm. The overnight culture was diluted in fresh LB to an A_600nm_ of 0.05 (± 0.01) and 300 μl of this diluted LESB58 culture was added to 30 ml ASM. The ASM cultures were incubated at 37°C for 7 days at 50 rpm. Where appropriate, sub-inhibitory concentrations of either ceftazidime (0.125 μg/ml), colistin (1 μg/ml), meropenem (2 μg/ml), tobramycin (2 μg/ml) or azithromycin (0.25 μg/ml) were added to the ASM. The minimum inhibitory concentrations were of ceftazidime 8 μg/ml, tobramycin 16 μg/ml, ciprofloxacin 168 μg/ml, colistin 8 μg/ml, meropenem 16 μg/ml, and azithromycin 16 μg/ml. Sub-inhibitory concentrations were determined by testing the growth of *P. aeruginosa* LESB58 exposed to a dilution series of these antibiotics in ASM. The antibiotics were then tested at 8, 16, 32, 64-fold below the minimum inhibitory concentration, and the antibiotic concentration used was the highest that did not affect the growth rate in ASM. Therefore, the sub-inhibitory concentration of each antibiotic was the highest concentration of antibiotic that still allowed culture absorbance readings similar to that of the negative control (LESB58 grown in the absence of antibiotics). In addition, as a control LESB58 was cultured in ASM without antibiotics for 7 days. The experimental design for analysing *P. aeruginosa* LESB58 populations cultured in ASM, with and without antibiotics, is shown in Figure 4. Visible biofilms had formed by day 2 of LESB58 culture in ASM and increased in size by day 3. There were no visible changes in the biofilm mass between day 3 and day 7 of incubation. There were no visible differences between the biofilms formed in the ASM in the presence of the various antibiotics, compared to the biofilms formed in ASM without antibiotics. Following the 7 day incubation, the ASM was treated with Sputasol (Oxoid, Basingstoke, UK) in a ratio of 1:1 and incubated for 30 min at 200 rpm and at 37°C. Sputasol has been used in previous studies to liquefy the biofilms formed in ASM and to release the *P. aeruginosa*[9,55,57]. The sputasol-treated cultures were serially diluted and grown on Columbia agar (Oxoid). Columbia agar has been used in previous studies to culture *P. aeruginosa*[7,57]. Additionally, the widely-used Miles and Misra method was performed to determine the numbers of bacterial CFU/ml [58]. Following overnight growth, 40 isolates per 30 ml volume of ASM were randomly selected. The 40 isolates selected from each 30 ml volume of ASM did not represent technical replicates. The experiments involving culture of LESB58 in ASM (with or without antibiotics), and the subsequent analysis, were performed in triplicate. Therefore, 120 isolates from each experimental and ASM control group were analysed using various phenotypic and genotypic tests. Furthermore, to demonstrate the absence of extensive diversity in the LESB58 populations that seeded the ASM cultures, we assessed the phenotypic and genotypic properties of LESB58 following culture in LB for 18 hours (40 isolates were selected from three LESB58 cultures in LB).

### Genotypic tests

The earliest available LES isolate, LESB58 (from 1988), has been genome sequenced and it contains 5 GIs (including LESGI-5) and 5 complete prophages (including LES prophages 2 and 5) within its accessory genome [56]. PCR assays were used to screen for LES prophage 5, LES prophage 2 and LESGI-5 (Table 3). PCR amplifications were carried out in a volume of 25 μl. Each reaction contained 1.25 U GoTaq polymerase (Promega, Southampton, UK), 1x Green GoTaq Flexi buffer (Promega), 300 nM of each oligonucleotide primer (Sigma-Genosys, Haverhill, UK; Table 3), 2.5 mM MgCl_2_ (Promega), 100 mM nucleotides (dATP, dCTP, dGTP, dTTP; Bioline) and 1 μl DNA from boiled suspensions of colonies. Amplification was carried out for 30 cycles of 95°C (1 min), the annealing temperature (2 min) and 72°C (2 min), after which, a final extension step of 72°C for 10 min was carried out. Electrophoresis of PCR products was performed using a 1% (w/v) agarose gel at 100 V for 1 h.

**Table 3:** Oligonucleotide primers used in this study

**Name**	**Sequence (5′-3′)**	**Size (bp)**	**Annealing temperature (°C)**	**Target gene**	**Reference**
LESD3cIF	ATGAAAAAGCCCGTAAGA	490	55	LES prophage 5 *c*I repressor gene	[13]
LESD3cIR	GCCATTCCCGCTTAAAAG				
LES1F	TCGGCGTAATGTCCTCTA	392	68	LES prophage 2	[59]
LES1R	TGAAGCCGACGATGGAAG				
PS1F	ACAGAATATTCGAAGCAG	338	58	LES genomic island-5	[59]
PS1R	ACAAGAGCCTAACACCAC				

### Phenotypic tests

The phenotypic tests used are those described previously for our study of isolates from CF patients [9]. Colony morphology was assessed on Columbia agar. Auxotrophy was investigated by testing the ability of isolates to grow on glucose M9 media. Hypermutability was assessed by determining the spontaneous mutation rates on LB agar containing rifampicin (Sigma-Aldrich; 300 mg/ml) following overnight growth in LB broth, as previously described [45]. Overproduction of pyocyanin was detected and measured using pre-determined cut-off values [60]. Isolates were classified as overproducers of pyocyanin when the culture supernatant had an absorbance greater than 0.1 at 695 nm, following overnight growth in 5 ml LB broth at 200 rpm. The sensitivity and resistance profiles of the individual isolates to antibiotics commonly used to manage CF infections (ceftazidime, colistin, meropenem, tazobactam/piperacillin, ciprofloxacin and tobramycin; all from Oxoid) were determined using the disk diffusion method. The sizes of the zones of inhibition (mm) were recorded, and compared to the zone sizes generated from replicates of *P. aeruginosa* LESB58 used as controls (n = 120). Zones sizes that were outside the range (either above or below) that was observed for the replicates of LESB58, were reported as being different from the founder (LESB58). The following amounts of antibiotics were present in the disks: 85 mg tazobactam/piperacillin, 10 mg meropenem, 10 mg tobramycin, 5 mg ciprofloxacin, 30 mg ceftazidime and 25 mg colistin sulphate, as recommended by British Society for Antimicrobial Chemotherapy guidelines [37].

### Defining a haplotype

In this study, a haplotype was defined as a specific combination of phenotypic and genotypic traits. Diversity was displayed using the eBurst algorithm [61], which produces a diagrammatical representation of the diversity within a bacterial population, and can be used to show where the founder haplotype (LESB58) diversifies to produce a cluster of closely related haplotypes. To obtain an eBurst diagram, each phenotypic and genotypic trait was assigned a numerical code and, therefore, each haplotype had a specific combination of numerical values [9]. The eBurst algorithm was used to compare the numerical profiles of each haplotype, in order to determine relatedness between haplotypes. Isolates characterised as haplotype number one had the same trait values as *P. aeruginosa* LESB58 (“The Founder”). These traits were a green non-mucoid colony morphology on Columbia agar, over-production of pyocyanin, resistance to ceftazidime and an auxotrophic, non-hypermutable phenotype. Haplotypes one position away from the founding haplotype on the eBurst diagrams differed in one trait from LESB58, and isolates two positions away from the founding haplotype on the eBurst diagram differed in two traits. This method of analysing *P. aeruginosa* haplotypes has been published previously by Mowat *et al.*[9].

### Statistical analysis

A generalised linear model with a negative binomial error distribution was used to test whether the number of novel haplotypes was differed between ASM and ASM plus antibiotic treatments, with significance assessed using a likelihood ratio test. Haplotype diversity was calculated as the probability of two randomly picked clones being the same haplotype based on the haplotype frequencies within a sample (equivalent to the Simpson’s Index) and analysed in a linear model following a logistic transform. Hierarchical analysis of variance was performed using the ade4 package in R [62] in order to estimate the population differentiation between treatment groups, between populations within treatment groups and between clones within populations.

## Abbreviations

ASM: Artificial sputum medium; AZT: Azithromycin; CAZ: Ceftazidime; CF: Cystic Fibrosis; CT: Colistin; GI: Genomic Island; LES: Liverpool Epidemic Strain; LB: Luria Bertani; MEM: Meropenem; ND: Not determined; QS: Quorum Sensing; TOBI: Tobramycin.

## Competing interests

The authors declare that they have no competing interest.

## Authors’ contributions

EAW carried out the experimental studies and helped draft the manuscript. JLF participated in the design of the study. SP performed some of the statistical analysis and helped draft the manuscript. MAB gave intellectual input on the statistical analysis and helped draft the manuscript. CW conceived the study, participated in its design and coordination and helped draft the manuscript. All authors read and approved the final manuscript.
